# Indigenous health: designing a clinical orientation program valued by learners

**DOI:** 10.1186/s12909-017-1019-8

**Published:** 2017-10-05

**Authors:** Tania Huria, Suetonia Palmer, Lutz Beckert, Cameron Lacey, Suzanne Pitama

**Affiliations:** 1 Māori and Indigenous Health Institute, University of Otago, 2 Riccarton Ave, Christchurch, 8140 New Zealand; 2 Department of Medicine, University of Otago, 2 Riccarton Ave, Christchurch, 8140 New Zealand

**Keywords:** Indigenous, Medical education, Cultural competency

## Abstract

**Background:**

Indigenous health programs are seen as a curriculum response to addressing health disparities and social accountability. Several interrelated teaching approaches to cultural competency curricula have been recommended, however evidence of the impact of these on learner outcomes including engagement and self-reported competencies is limited. We aimed to explore undergraduate medical student perspectives of an indigenous health orientation program to inform curriculum strategies that promote learning and development of clinical skills.

**Methods:**

We analyzed quantitative and qualitative student evaluations (*n* = 602) of a three-day immersed indigenous health orientation program between 2006 and 2014 based on Likert-scale responses and open-text comments. We conducted a thematic analysis of narrative student experiences (*n* = 426).

**Results:**

Overall, 509 of 551 respondents (92%) rated the indigenous health orientation program as extremely or highly valuable and most (87%) reported that the course strongly increased their interest in indigenous health. The features of the clinical course that enhanced value for learners included situated learning (*learning environment; learning context*); teaching qualities (*enthusiasm and passion for Māori health; role-modelling*); curriculum content (*re-presenting Māori history*; *exploring Māori beliefs, values and practices; using a Māori health framework in clinical practice*); teaching methodologies *(multiple teaching methods; simulated patient interview)*; and building relationships with peers (*getting to know the student cohort; developing professional working relationships*).

**Conclusions:**

Undergraduate medical students valued an indigenous health program delivered in an authentic indigenous environment and that explicitly reframed historical notions of indigenous health to contextualize learning. Content relevant to clinical practice, faculty knowledge, and strengthened peer interactions combined to build learner confidence and self-reported indigenous health competencies. These findings suggest empirical evidence to support a curriculum approach to indigenous health teaching that enhances clinical learning.

## Background

Indigenous peoples have experienced marked and persistently worse health outcomes than non-indigenous peoples since colonization [1–3]. On average, life expectancy among indigenous peoples is nearly ten years less than non-indigenous populations around the world. The burden of communicable and non-communicable disease among indigenous peoples is highly disproportionate [4]. Empirical evidence suggests that clinicians and institutional healthcare practices (such as racial bias and lower rates of referral and preventative treatment) sustain health inequities [5, 6]. In response, cultural competence curricula have emerged and become mandated within undergraduate and post-graduate health education. National accrediting bodies have required medical schools to include indigenous health as core curriculum content. However, evidence suggests that cultural competency programs are less valued by learners than other subjects [7] and the impact of cultural competency programs on health disparities remains to be fully realized [8–11]. Clinicians are aware of the impact of ethnicity on worse health outcomes, however lack confidence to meet their patients’ specific needs [12].

The specific pedagogies of indigenous health teaching programmes that maximize clinical learning and learner engagement are incompletely understood. Kripalani and colleagues have recommended several teaching approaches within cultural competence education including an emphasis on practical skills teaching, interactive methods, sequential rather than single curriculum encounters, framing cultural competence as a ‘real science’, and visible commitment by faculty leaders [13]. Despite these recommendations, learner experiences of specific teaching strategies within indigenous health curricula have not been fully explored. The evidence supporting the impact of indigenous health education on medical student skills is sparse [14].

Learner perspectives can inform curriculum design and guide learner-appropriate pedagogy within indigenous health programs. This study aimed to explore medical student experiences and perceptions of an orientation course for indigenous health competencies during undergraduate medical training.

## Methods

We conducted a quantitative and thematic analysis of medical student evaluations of an introductory teaching program for indigenous health (hauora Māori) at a single medical school campus in New Zealand. The study was conducted using kaupapa Māori methodology (a methodology guided by indigenous principles including design, analysis, and reporting by Māori clinical teachers and clinicians) [15]. Kaupapa Māori methodology provides theoretical foundations that support the development of a research process that promotes Māori researchers, Māori pedagogies, Māori research leadership [15].

### Setting

This study was conducted at the University of Otago, Christchurch, in New Zealand. The undergraduate medicine curriculum is six years including an initial health sciences year, two pre-clinical years, and three clinical years at one of three clinical schools (Dunedin, Christchurch, and Wellington). The clinical curriculum at the Christchurch campus of the Otago Medical School includes a two-week clinical orientation to the clinical learning environment, and includes a three-day and two-night immersed indigenous health orientation to the indigenous health curriculum (hauora Māori). Medical students attend the indigenous health orientation program at an indigenous meeting place (marae). During the indigenous health orientation, the students are introduced to the Meihana model, a clinical assessment framework [16], and the Hui Process, a framework for the doctor-patient relationship with Māori [17]. A core group of Māori and non-Māori clinicians including a head of department of academic medicine (author LB) and Associate Dean Māori (author SGP) have collaboratively developed and led the teaching indigenous health orientation for over ten years.

### Data collection

The indigenous health orientation program was nested within a two-week clinical orientation program to the hospital and primary care settings. Anonymous written evaluations of the two-week clinical orientation were requested from medical students attending the course between 2006 and 2014. Students completed two separate evaluations. The first asked about views and perceptions of the overall clinical orientation program. The second explored student responses to the indigenous health orientation program. The surveys were administered independently of teaching staff to encourage open and honest student feedback. Surveys included both Likert-scale ratings and narrative responses to open-ended questions. The purpose of the evaluations was to determine the perceived usefulness of the indigenous health orientation program to student clinical practice. The qualitative component aimed to identify factors that contributed to students’ perceptions and experiences. Surveys were administered annually between 2006 and 2010, but were not completed in 2011 because of the Canterbury earthquakes, due to changes in University student evaluation policy students were invited to participate in the indigenous health orientation program evaluation biannually until 2014. Student participation was varied in the quantitative surveys namely due to student attendance at the time of the survey administration.

### Data analysis

We summarized student characteristics and scaling responses descriptively. Likert-scale scores for sub-courses in overall orientation program, including the indigenous health orientation, were compared using a Friedman’s test to account for multiple related samples.

Narrative student responses were imported into NVivo version 10 (QSR International Pty Ltd., 2014) software for organization and analysis. First author (TH) reviewed all original surveys and responses. The senior author (SGP) performed the initial qualitative data analysis. Kaupapa Māori methodology was used to guide the data analysis to ensure that it captured the student experiences and perceptions of the clinical program. An inductive approach was used to identify themes that emerged from the content. In the first cycle of coding, descriptive analysis was used to organize the data, which drew on a word or short phrase to ‘describe’ the basic topic or content of the data [18]. In the second cycle of coding, theoretical coding was used to reorganize, further refine, and ‘make sense’ of the data [18]. Theoretical coding was used to draw together similar codes and categories to ensure the ‘overall’ story (theoretical direction) best represented a cohesive participants’ narrative and identify theoretical sufficiency within each theme [19]. Further discussions about the emerging themes were held between all authors (who were all lecturers in the indigenous health orientation program) to ensure the thematic analysis fully captured and explained the student responses. A thematic schema was generated to conceptualize the themes and subthemes.

## Results

### Participants

Of the 550 students in the clinical orientation program who were invited to complete a survey, 351 (64%) offered complete responses. The indigenous health orientation survey was completed by 602 students (94%) of the 641 students invited to participate. Not all respondents answered all questions. The response rates for individual questions in the indigenous health orientation survey ranged between 88.3% and 91.3%. Overall, 426 respondents provided 860 qualitative comments for the three open-ended questions; 390 replied to the question “What was the best aspect of the course?”; 262 replied to the question “What improvements could be made?”; and 208 provided “Other comments”.

### Quantitative analysis

**Fig. 1:**
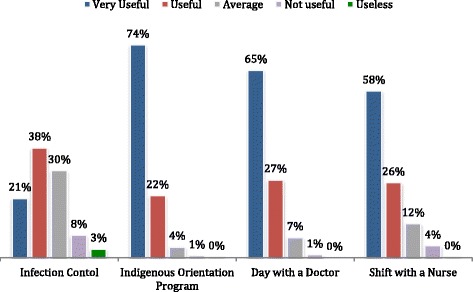
Student evaluations of modules within the overall clinical orientation program

Five hundred nine of 551 respondents (92%) rated the indigenous health orientation program via the indigenous health orientation program evaluation as extremely or highly valuable, while most (87%) reported that the course strongly increased their interest in Māori health (Table 1). The summary evaluations of four major components in the clinical orientation program including the indigenous health orientation are shown in Fig. 1. 286 of 298 (96%) students that completed the evaluation rated the indigenous health orientation program as “useful” or “very useful”, while the proportions reporting “useful” or “very useful” ratings for the “Shift with the Nurse”, “Day with a Doctor”, and Infection Control learning sessions were 84%, 92%, and 59% (*p* value for differences <0.001). The median scores for each curriculum component represented by a 5-point scale (1 = very useful and 5 = useless) were; Māori health (2.04), Day with a Doctor (2.23), Shift with a Nurse (2.40), and Infection Control (3.33) (*p* < 0.001).

**Table 1:** Student survey responses to indigenous health orientation program

Survey questions	1 = Extremely Useful (%)	2 = Very Useful (%)	3 = Average (%)	4 = Not very Useful (%)	5 = Useless (%)	Response Rate (%)
How valuable do you consider this course has been for you?	49.4	43	5.4	1.6	0.5	91.5
Did this course increase your interest in the subject matter?	42.4	45	9.8	2.0	0.7	89.7
Rate the value of the field trip as a learning experience.	62.9	30.3	5.5	1.1	0.2	90.4
To what extent have you reached a deeper understanding of this clinical area?	27.3	61.9	9.5	1.4	0.0	89.9
How much did this course challenge you to think?	38.7	46.4	12.9	1.4	0.5	88.3
Did you learn to value new viewpoints because of this course?	63.2	43	6.3	1.4	0.3	88.3

### Thematic analysis

**Fig. 2:**
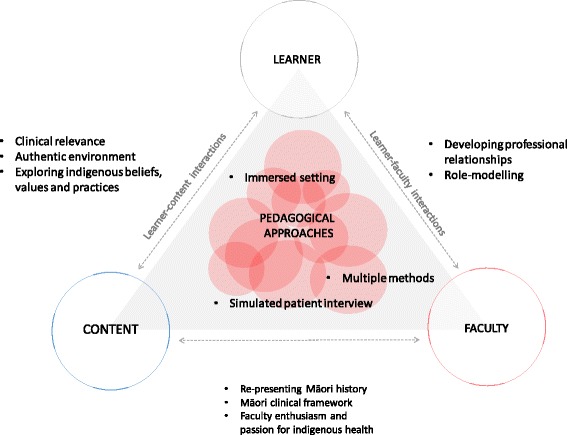
Thematic schema. The indigenous health curriculum was conceptualized as an interaction between learner, faculty, and indigenous health, which defined appropriate and flexible pedagogical techniques embedded within an indigenous world view. Learners experienced multiple teaching strategies that were specific to indigenous health and were aimed at maximizing clinical relevance. Learners valued course content delivered in an authentic environment and that explicitly reframed historical notions of health determinants. Relevant content, faculty knowledge, and strengthened peer-support and relationships combined to enable learners to interact with simulated indigenous patients in a way that built clinical confidence and self-reported competencies

Inductive analysis of narrative survey responses identified five key themes (and related subthemes) to describe the teaching approaches that students valued in the indigenous health program: situated learning (*learning environment; learning context*); teaching qualities (*enthusiasm and passion for Māori health; role-modelling*); curriculum content (*re-presenting Māori history*; *exploring Māori beliefs, values and practices; using a Māori health framework in clinical practice*); teaching methodologies *(multiple teaching methods; simulated patient interview)*; and building relationships with peers (*getting to know the student cohort; developing professional working relationships*). The successful teaching approaches were conceptualized as interactions between learners, faculty, and content to generate pedagogies that maximized contextualization of indigenous health through history, provided an authentic environment to amplify respect for indigenous perspectives, made content relevant to clinical practice, and strengthened peer-peer interactions to increase experiential learning support (Fig. 2).

### Situated learning

#### Learning environment

Students valued learning and living at a marae (indigenous meeting place) as an authentic environment in which to learn, and which motivated them to engage with the course content more deeply. Students valued staying at the marae for three days and two nights. Utilising the marae as a learning environment enabled students to experience specific traditional protocols that broadened their understanding and familiarity of Māori culture and enabled them to see its relevance within a healthcare setting.

*“Change of environment. Experiencing the Māori culture is a very effective way of getting an understanding. This is so much better than just having lectures in school. Just staying in the marae makes me ‘feel’ the culture, not just learning.” (P28, 2007)*

#### Learning context

Students commented that they appreciated the privilege of being in an immersed environment that was picturesque, had historical meaning within New Zealand history, and assisted them to understand the connection between indigenous peoples and their land and place. Students valued the timing of the course being at the beginning of their clinical practice before they interacted with Māori patients and families, as they saw that being in an immersed setting allowed them to develop a sense of respect and understanding of the relevance of indigenous status to health.

*“the atmosphere in the marae is so different to the classroom atmosphere and I think the field trip experience definitely helped me understand abstract concepts of how the community values their land and marae.” (P23, 2010)*

### Teaching qualities

#### Enthusiasm and passion for Māori health

Students valued the qualities shown by faculty staff who facilitated and contributed to the Māori health programme. In particular, students described the staff’s enthusiasm for teaching about Māori health. The role modelling from staff motivated students to learn, explore the course content, and increased their confidence to participate in learning.

*“The teachers, they had an amazing passion for Māori Health and a great understanding of it which they conveyed completely to us. Having passionate, knowledgeable teachers really encourages you to remember or take an interest in what they are saying.” (P1, 2008)*

This enthusiasm also aided greater learner comprehension of the curriculum content. Students reflected that the teachers’ passion for the curriculum helped to create a safe learning environment in which they felt able to ask questions and explore new and difficult concepts.

*“Having fantastic teaching staff, every one of them, making it all very interesting for the first time. Bringing it to life and making it all very relevant. Really enjoyed their enthusiasm and passion which was definitely very infectious.” (P34, 2008)*

#### Role modelling

Students valued being taught by both Māori and non-Māori clinicians who were enthusiastic about working with Māori patients, who shared personal anecdotes, and who supported students learning as role models for working with Māori patients and families. Students appreciated the time that staff took to show how they had successfully incorporated the Māori health framework into their own clinical practice and how this had helped them to provide better health care for Māori patients and families.

*“It’s so refreshing to see enthusiastic teachers who take Māori Health seriously – so we did too. Clinical case scenarios were really valuable – realistic situations which were cleverly constructed to help us learn the skills we had been taught earlier.” (P39, 2007)*

### Curriculum shaped by cultural knowledge

#### Re-presenting Māori history

Students valued being challenged about their existing assumptions and stereotypes about indigenous patients and communities. They specifically responded positively to a lecture that deconstructed the process of colonization, drawing on art history and anthropological and epidemiological evidence to re-present colonization from an indigenous perspective. Students critically reflected on how dominant and influential perspectives had shaped their understanding of indigenous cultures and health experiences. The lecture content had also helped them to see a broader ‘purpose’ to studying Māori health as part of their medical education, and better perceive their role in contributing to Māori health outcomes.

*“Understanding the Māori viewpoint on healthcare and disparities in healthcare – I had always saw it as Māori wanting more, not wanting equal.” (P1, 2008)*

#### Exploring Māori beliefs, values and practices

Students also valued the course content that focussed on the Māori world (including cultural protocols, beliefs, and language) that they could use to facilitate engagement with patients during clinical encounters. Some students also highlighted that they would have liked more of this specific content as they felt for some areas they had only just ‘touched lightly’ on this knowledge and that they needed to know more.

*“It is really good to learn and know some of their culture, beliefs and values which I believe would be valuable for me as medical student.” (P10, 2007)*

#### Using a Māori health framework within clinical practice

Students valued being taught specific clinical Māori health frameworks and having opportunity to apply these to their clinical practice. Students mentioned how the course content had positively impacted them to engage more confidently with Māori patients and families and gain relevant clinical competencies.

*“Learning how to apply the Meihana model to the Calgary-Cambridge model – I feel far better equipped now to work with Māori patients.” (P36, 2010)*

*“I really enjoyed learning the Hui process with respect to engaging future patients and establishing relationships in a clinical setting.” (P20, 2012)*

### Teaching methodologies

#### Multiple teaching methods

Student comments highlighted that they valued the use of multiple teaching methods. Students expressed an appreciation for the variation in teaching methodologies that included lectures, small group work and workshops, which helped them to engage with the content. Students also reported that different teaching methods and environments encouraged high levels of interaction between each other and with the teaching team. This increased student interest in the course content.

*“The balance between interaction/participation, small group and lecture style teaching. The most valuable part of the content was the workshops and the skills they instilled in us for conducting history taking with a Māori patient.” (p41, 2007)*

#### Simulated patient interview

Students particularly valued the opportunity to participate in a simulated patient interview at the end of the course and to practice the clinical skills within a small group of peers. Students reported that this teaching method helped them to consolidate the learning and supported them to apply strategies they had been taught within a clinical scenario. Students reported that they enjoyed learning from watching their peers, and valued the feedback given by their peers and teachers as safe and supportive.

*“The simulated clinical scenario was really good to put what we had learnt into practice, a good way to go over what we had learnt. It also helped me feel more comfortable about using it in the future.” (P1, 2014)*

Students recognised the perceived authenticity of the Māori patient (actor/community member) within the case scenario, and expressed how ‘realistic’ the scenario felt to them. Students noted that the simulated patient session was challenging, however reflected that it assisted them to navigate through components of the Māori health frameworks.

*“Learning how to apply the Meihana Model to the Calgary-Cambridge Model – I felt far better equipped to work with Māori patients” (P10, 2010)*

### Building relationships with peers

#### Getting to know the student cohort

Some students identified learning in small groups of peers (compared to their pre-clinical years 2 and 3) as a key factor in helping them to get to work more collaboratively with each other and staff to complete learning tasks. Students highlighted that “getting to know their peers” was a highly-appreciated component of the programme.

*“Getting to know new people and working together with them towards the same goal and involvement of everyone in the activities and exercises.” (P2, 2007)*

#### Developing professional working relationships

Students described that the small group activities involved a lot of interactive discussion and required open and honest interactions between and among students to create a safe learning environment. These small-group work activities were highly valued by the students because allowed them to explore new content areas with peer support and develop collegial bonds before entering their clinical years.

*“Learning with classmates I hadn’t known, trusting new people/unfamiliar faces to share answers, information – bonding!” (P2, 2010)*

## Discussion

This study describes the characteristics of an indigenous health program that increase learner engagement with content and enhances their development of clinical competencies in care with indigenous patients. Overall, indigenous and non-indigenous faculty partnerships facilitated the reframing of conventional notions of indigenous health to contextualize learning. This contextualization motivated students to consider the course as relevant to their clinical training. Students valued the situational learning environment (marae) because it offered them authenticity and connection with indigenous values, and that it was a safe environment to move beyond a conceptual approach of cultural education. Immersed learning also strengthened peer-to-peer relationships. Introduction of an explicit clinical framework (the Meihana model [16]) to engage with indigenous patients together with experiential small-group work resulted in confident practice with simulated indigenous patients. This study provides empirical evidence for the elements of an indigenous health program that are most valued by learners to increase their self-confidence and clinical competencies.

This study indicates that incorporating the specific teaching strategies within a cultural competence program, as previously proposed by Kripalani and colleagues [13], can help students to value an indigenous health curriculum at levels similar to mainstream course content. These findings provide empirical evidence of the core elements of curriculum development that might enable clinicians to overcome their inability to enact culturally-sensitive care [20]. This is particularly relevant, as health practitioners frequently express concern about implementing specific cultural competencies during clinical encounters. In addition, this study offers specific information for curriculum design in indigenous health, in an area of medical education in which existing evidence is sparse ([14], Pitama, S: As Natural as Pathology, unpublished thesis). Our findings indicating that a cultural competency curriculum can be designed to increase learner-reported engagement and competence counters previous investigations that have concluded that both students and clinical teachers consider existing cultural competency programs to have insufficient clinical applicability [21].

**Fig. 3:**
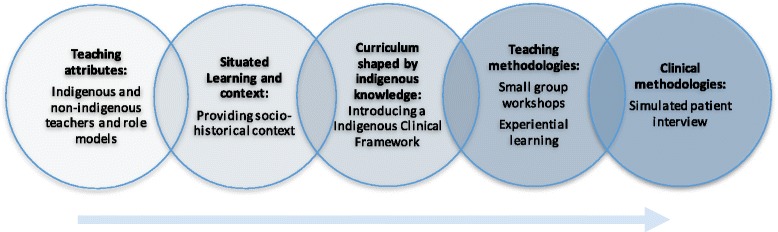
Schema describing proposed development sequence for design of an indigenous health curriculum. This figure is a representation of potential key aspects to designing an indigenous health orientation program as identified by learners. The thematic analysis of learner responses is interpreted to suggest a required sequence of steps for curriculum development. Indigenous and non-indigenous faculty members and role models reframe indigenous health within a historical context and indigenous setting to enhance relevant and contextualize disparity. Students learn an indigenous health framework [16, 17] in small groups with experiential learning approaches before interviewing a simulated indigenous patient

Existing evidence suggests indigenous health education is an emerging pedagogy, and studies have raised concerns about the quality of teaching and assessment of indigenous health [14]. Institutional and educational barriers may exist that impact on the value that students and clinical teachers place on indigenous curricula. These barriers include a focus on science rather than humanities, and a lack of opportunity for indigenous faculty leadership, resulting in inadequate resourcing of indigenous content within health education. The work outlined by this research provides an educational schema to support the development of learners and faculty awareness of indigenous health disparities and a way to respond that engages training clinicians. The program described in this study has identified methods that enable students to value an Indigenous health curriculum as an equal part of their medical education. The program provides a framework that addresses both the institutional and educational barriers identified by similar studies such as limited institutional support and/or understanding of the critical importance of an Indigenous health curricula to support clinical training [22] (Fig. 3). Future work would include exploration of the impact of the curriculum on objective measurements of clinical competencies.

Despite the strengths of this study (multiple learner cohorts over several years; large sample size; high response rate; inductive analysis of themes with researcher triangulation; a kaupapa Māori approach [15]), there are limitations that need to be considered. First, we did not include detailed interviews with respondents to explore the concepts in more detail. Second, we drew on narrative survey responses that were intended for course evaluation and were analyzed *post-hoc*. Third, it is possible that responses were received from learners who had particularly positive or negative responses to the curriculum and thus may not be generalisable to overall student experience. Finally, this study evaluated the teaching interventions and did not explore the impact of the curriculum on clinical interactions with indigenous patients.

## Conclusions

This study reports the teaching structure of an indigenous health program that is highly valued by learners as relevant to their training and clinical competencies. Awareness of historical and contemporary indigenous perspectives to contextualise knowledge together with multiple pedagogical approaches, faculty role-modelling, and peer interaction during teaching support learner engagement and skill development can build a curriculum that increases learner confidence. Future research to explore the impact of indigenous health curricula on clinician competencies and patient care would support the ongoing development of medical education responses to indigenous health disparities.
